# Supplementary material for: The theta paradox—delayed reduction of frontal midline theta following downregulation neurofeedback training
Source: Front Hum Neurosci. 2026 Jul 22;20:1806951. doi: 10.3389/fnhum.2026.1806951 (PMC13438158; doi:10.3389/fnhum.2026.1806951)
Supplement: Supplementary file 1 [file Image_1.PDF]

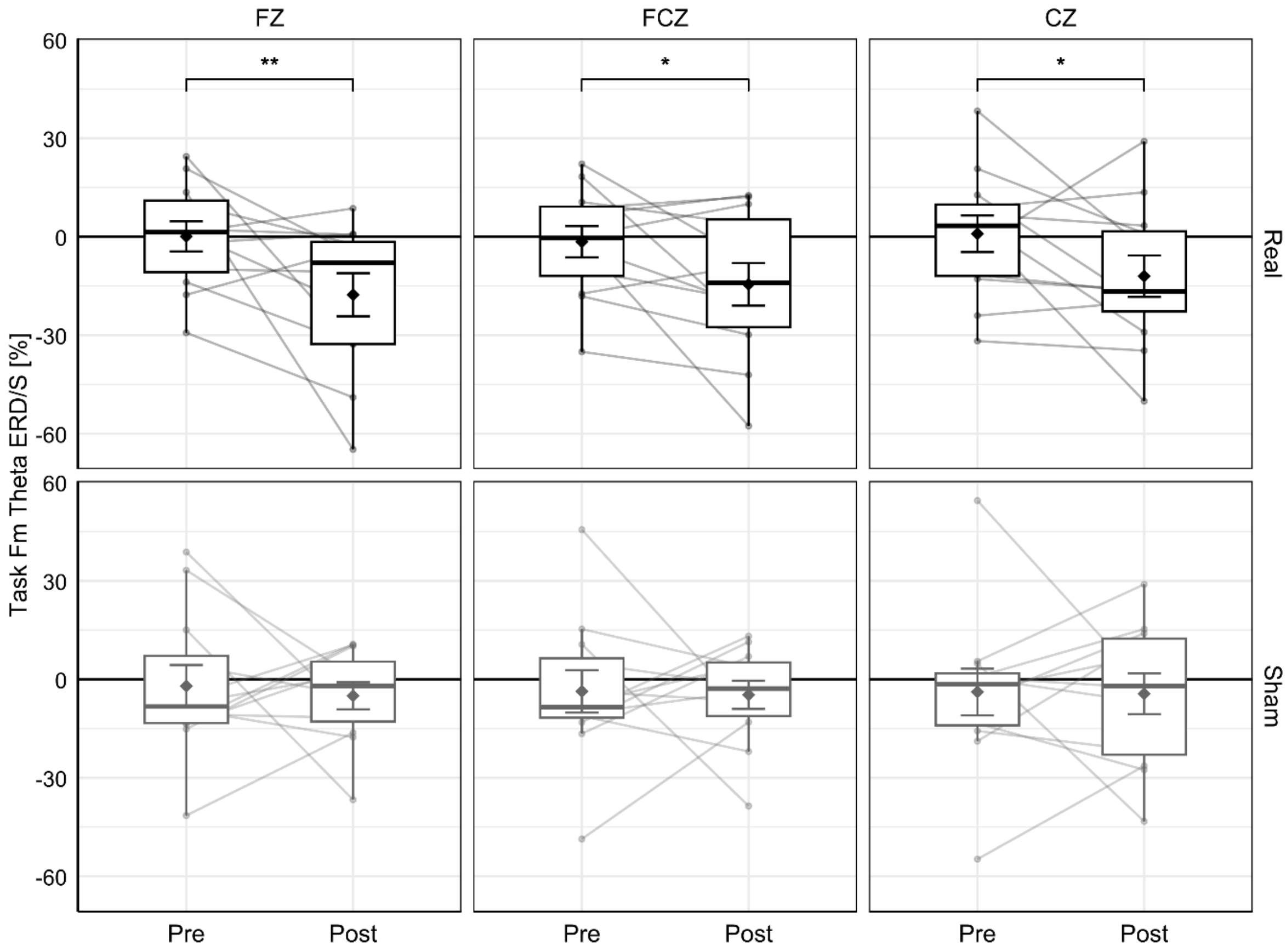

Fig. S1 Results for the analysis of Task Fm Theta ERD/S separated by ROI electrodes. Pre/post differences of individual participants are represented by line-connected dots. Means and standard errors are represented within boxplots by diamond symbols and error bars. Significant pre/post differences, as determined through LMM analysis, are indicated with asterisks (*: $p < .05$; **: $p < .01$).
