# Supplementary material for: The theta paradox—delayed reduction of frontal midline theta following downregulation neurofeedback training
Source: Front Hum Neurosci. 2026 Jul 22;20:1806951. doi: 10.3389/fnhum.2026.1806951 (PMC13438158; doi:10.3389/fnhum.2026.1806951)
Supplement: Supplementary file 2 [file Table_1.DOCX]

Table S1 Deviations from preregistration

| **Method:** | **Prereg:** | **Article:** |
| --- | --- | --- |
| Dependent variable | … For the analysis of fm-theta ERD/S during the task, a pre-stimulus “Baseline” phase (2 s visual fixation) will act as a reference to the “Video” phase (last 3 s before the end of each video stimulus), as well as the “Freeze” phase (0.5 s freeze of last video frame immediately before response), which are the main timeframes of interest. … | In the present article the task has been renamed from “tactical decision-making task” into “volleyball setting anticipation task” (VSAT). As the definitions of specific phases of the task have been expanded over time, what was previously referred to as the “Video” phase was renamed to “Rally” phase in the present article, analogously to our previous article in which neurophysiological substrates of volleball anticipation were investigated (Kanatschnig et al., 2025). As these deviations are merely semantical, they did not impact our statistical analyses. |
| Analyses | … The analysis of behavioral performance and brain activity during the tactical decision-making task, will be conducted by means of four separate mixed-design ANOVAs. These will consist of Group and Time as independent variables and task accuracy and task response time, as well as fm-theta ERD/S, during the Video and Freeze phases, as dependent variables, including Holm-corrected post-hoc t-test comparisons where necessary. … | With the use of LMM we deviated from our preregistered methods, in which we originally planned to use mixed-design ANOVA. However, the use of a more comprehensive LMM structure can be deemed an improvement of test severity, which makes this deviation an overall beneficial change regarding statistical rigor (Lakens, 2024). In the preregistration we also specified to investigate the Freeze phase of the task. Fm Theta ERD/S during the Freeze phase was analyzed analogously to the Rally phase. However, due to a lack of relevant additional information, we decided to omit the results of the Freeze phase in the main article, yet they are available for inspection through the provided online materials (<https://osf.io/bu924/>; Kanatschnig et al., 2024). |
| *Note*. Methods that were preregistered for this study at the Open Science Framework (<https://osf.io/hzg9j>), for which the actual implementation in the article deviated are described under Prereg. Implemented procedures are described under Article. | | |
