# Supplementary material for: The theta paradox—delayed reduction of frontal midline theta following downregulation neurofeedback training
Source: Front Hum Neurosci. 2026 Jul 22;20:1806951. doi: 10.3389/fnhum.2026.1806951 (PMC13438158; doi:10.3389/fnhum.2026.1806951)
Supplement: Supplementary file 3 [file Table_2.DOCX]

Table S2 Comparative statistics for main sociodemographic and control variables

| **Variables:** | **Real (*n* = 12):** | **Sham (*n* = 12):** | **Statistics:** | | | |
| --- | --- | --- | --- | --- | --- | --- |
| **Categorical** |  |  | ***df*** | ***χ^2^*** | ***p*** | ***V*** |
| Sex (female/male) | 8/4 | 8/4 | 1 | 0 | .999 | .00 |
| “Do you currently play actively in a volleyball club?” (no/yes) | 12/0 | 12/0 | 1 | 0 | .999 | .00 |
| “Do you play volleyball in your free time?” (no/yes) | 6/6 | 11/1 | 1 | 3.23 | .072 | .37 |
| “Are you currently attending one or more volleyball courses at the University Sports Institute (USI)?” (no/yes) | 12/0 | 12/0 | 1 | 0 | .999 | .00 |
| “Apart from volleyball, do you play sports regularly?” (no/yes) | 2/10 | 1/11 | 1 | 0 | .999 | .00 |
| **Continuous** |  |  | ***df*** | ***t*** | ***p*** | ***d*** |
| Age in years | 24.00 (±5.24) | 26.33 (±3.47) | 19.1 | -1.29 | .214 | -0.53 |
| “On average, how many hours a week do you play volleyball? (Please provide an approximate estimate of the number of hours.)” | 0.43 (±0.69) | 0.08 (±0.29) | 14.7 | 1.59 | .132 | 0.65 |
| “Approximately how many years of volleyball experience do you have? (Please provide a rough estimate of the years.)” | 2.33 (±3.06) | 1.83 (±2.82) | 21.9 | 0.42 | .681 | 0.17 |
| “On average, how many hours per week do you exercise? (Please provide an approximate estimate of the number of hours.)” | 3.33 (±2.05) | 5.67 (±4.42) | 15.5 | -1.66 | .117 | -0.68 |
| EHI | 74.58 (±17.38) | 77.08 (±21.89) | 20.9 | -0.31 | .760 | -0.13 |
| *Note*. Absolute participant counts for respective answer options are presented for each categorical variable, while means and standard deviations are presented for continuous variables, within the respective group columns. Categorical variables were examined using *χ^2^*-tests of independence plus Cramer's *V* calculations, while continuous variables were examined using independent samples *t*-tests plus Cohen’s *d* calculations. Degrees of freedom for independent samples *t*-tests were adjusted by means of the Welch method (Welch, 1947); “EHI”: Edinburgh Handedness Inventory (Oldfield, 1971). | | | | | | |
