# Supplementary material for: The theta paradox—delayed reduction of frontal midline theta following downregulation neurofeedback training
Source: Front Hum Neurosci. 2026 Jul 22;20:1806951. doi: 10.3389/fnhum.2026.1806951 (PMC13438158; doi:10.3389/fnhum.2026.1806951)
Supplement: Supplementary file 4 [file Table_3.DOCX]

Table S3 Comparative statistics for visual analog scale variables

| **Variables:** | **Real (*n* = 12):** | **Sham (*n* = 12):** | **Statistics:** | | | |
| --- | --- | --- | --- | --- | --- | --- |
| **Visual analog scale (VAS)** |  |  | ***df*** | ***t*** | ***p*** | ***d*** |
| 1. “How difficult did you find it to predict the pass position before you completed the neurofeedback training?” (Not difficult at all/Very difficult) | 60.92 (±21.85) | 75.83 (±19.97) | 21.8 | -1.75 | .095 | -0.71 |
| 2. “How difficult did you find it to predict the pass position after you completed the neurofeedback training?” (Not difficult at all/Very difficult) | 52.67 (±19.54) | 65.00 (±26.57) | 20.2 | -1.30 | .210 | -0.53 |
| 3. “How difficult did you find it to raise the middle bar during neurofeedback training?” (Not difficult at all/Very difficult) | 50.83 (±31.97) | 68.75 (±24.64) | 20.7 | -1.54 | .139 | -0.63 |
| 4. “How difficult did you find it to keep the two outer bars as low as possible during the neurofeedback training?” (Not difficult at all/Very difficult) | 29.83 (±28.23) | 28.92 (±29.83) | 21.9 | 0.08 | .939 | 0.03 |
| 5. “How do you think your ability to regulate your own brain activity has changed over the course of the neurofeedback training?” (No improvement/Strong improvement) | 50.25 (±23.13) | 35.58 (±23.91) | 22.0 | 1.53 | .141 | 0.62 |
| 6. “How motivated were you to complete the prediction tasks and the neurofeedback training?” (Not motivated at all/Very motivated) | 81.92 (±14.35) | 81.75 (±13.42) | 21.9 | 0.03 | .977 | 0.01 |
| 7. “How has your motivation changed over the course of testing?” (Decreased significantly/Increased significantly) | 50.33 (±19.50) | 54.00 (±24.16) | 21.1 | -0.41 | .687 | -0.17 |
| 8. “How concentrated were you during the prediction tasks and the neurofeedback training?” (Not concentrated at all/Very concentrated) | 71.58 (±16.03) | 77.75 (±20.11) | 21.0 | -0.83 | .416 | -0.34 |
| 9. “How has your concentration changed over the course of testing?” (Decreased significantly/Increased significantly) | 50.42 (±24.70) | 52.33 (±26.09) | 21.9 | -0.19 | .855 | -0.08 |
| 10. “Were you able to recognize patterns in the game scenes during the prediction tasks that helped you with the tasks?” (Not at all/Very much) | 53.83 (±25.21) | 42.00 (±22.25) | 21.7 | 1.22 | .236 | 0.50 |
| 11. “How much do you think your previous knowledge of volleyball helped you with the prediction tasks?” (Not at all/Very much) | 17.42 (±14.18) | 21.58 (±21.77) | 18.9 | -0.56 | .585 | -0.23 |
| 12. “In the prediction tasks, did you feel more like guessing or knowing the answer?” (Guess/Know) | 39.83 (±23.98) | 34.33 (±25.21) | 21.9 | 0.55 | .590 | 0.22 |
| *Note*. Means and standard deviations for VAS variables are presented within the respective group columns. Labels for the respective low (0) and high (100) ends of each scale are provided with each VAS variable. VAS variables were examined using independent samples *t*-tests plus Cohen’s *d* calculations. Degrees of freedom for independent samples *t*-tests were adjusted by means of the Welch method (Welch, 1947). | | | | | | |
