# Supplementary material for: The theta paradox—delayed reduction of frontal midline theta following downregulation neurofeedback training
Source: Front Hum Neurosci. 2026 Jul 22;20:1806951. doi: 10.3389/fnhum.2026.1806951 (PMC13438158; doi:10.3389/fnhum.2026.1806951)
Supplement: Supplementary file 5 [file Table_4.DOCX]

Table S4 Subject-level statistics and neurofeedback strategies

| **Code:** | **Group:** | **NFT Fm Theta ERD/S [slope]:** | **Task Fm Theta ERD/S [diff %]:** | **Rest Fm Theta [diff log(µV²)]:** | **NFT strategies:** |
| --- | --- | --- | --- | --- | --- |
| 1 | Real | 0.20 | -62.48 | 0.60 | nothing/relax, concentration bar, internal speech, concentration body, motor imagery |
| 2 | Real | 3.41 | -9.89 | 0.10 | concentration bar, nothing/relax, internal speech |
| 3 | Real | -5.53 | 13.19 | -0.09 | concentration internal, visual imagination |
| 5 | Real | 16.20 | -45.19 | 0.05 | concentration bar |
| 6 | Real | -4.22 | -8.79 | -0.28 | visual imagination |
| 7 | Sham | -0.29 | -9.78 | 0.05 | motor imagery, nothing/relax, concentration bar |
| 8 | Sham | 6.06 | 23.88 | 0.07 | nothing/relax, concentration bar |
| 9 | Sham | -0.36 | 20.69 | -0.12 | concentration bar, internal speech, moving eyes up/down |
| 10 | Sham | -1.33 | 22.99 | -0.25 | motor imagery, concentration internal, internal speech, body tension/relaxation |
| 11 | Sham | -0.25 | 3.24 | 0.28 | concentration internal, internal speech, emotion |
| 12 | Sham | -1.81 | -4.81 | -0.07 | concentration internal |
| 13 | Real | 0.22 | 6.37 | 0.18 | concentration bar, visual imagination |
| 14 | Real | -3.24 | -2.92 | -0.03 | concentration bar |
| 15 | Real | -1.55 | 4.55 | -0.13 | visual imagination, internal speech |
| 16 | Real | 3.54 | -24.66 | -0.40 | concentration bar |
| 17 | Real | 0.93 | -32.24 | -0.35 | nothing/relax |
| 18 | Real | -3.82 | -7.53 | -0.38 | motor imagery, nothing/relax |
| 19 | Sham | -1.85 | -53.79 | -0.09 | concentration bar, internal speech |
| 20 | Sham | -0.25 | -49.67 | -0.07 | concentration bar |
| 21 | Sham | 4.15 | -8.69 | 0.47 | nothing/relax, motor imagery, slow breathing |
| 22 | Sham | 3.20 | 29.74 | 0.60 | visual imagination |
| 23 | Sham | -2.31 | -7.96 | 0.21 | slow breathing, concentration bar, body tension/relaxation |
| 24 | Real | 3.52 | -5.21 | 0.15 | concentration internal, emotion |
| 25 | Sham | -2.63 | 15.89 | -0.07 | concentration bar, visual imagination |
| *Note*. Neurophysiological statistics are presented for each participant (nr. 4 was excluded) alongside reported NFT strategies categorized by common content. Values for NFT Fm Theta ERD/S represent NFT slopes, while Task Fm Theta ERD/S and Rest Fm Theta ERD/S represent pre-/post-NFT differences. | | | | | |
